# Identification of novel major quantitative trait loci associated with selenium and zinc accumulation in *Aegilops tauschii*

**DOI:** 10.3389/fpls.2026.1892742

**Published:** 2026-08-12

**Authors:** Hong Chen, Guoqing Wang, Wenhui Tian, Shunzong Ning, Dengcai Liu, Lianquan Zhang, Bo Zhang, Tao Liu

**Affiliations:** 1 Key Laboratory of Adaptation and Evolution of Plateau Biota, Northwest Institute of Plateau Biology, Chinese Academy of Sciences, Xining, China; 2 Qinghai Province Key Laboratory of Crop Molecular Breeding, Xining, China; 3 University of Chinese Academy of Sciences, Beijing, China; 4 State Key Laboratory of Crop Gene Exploration and Utilization in Southwest China, Sichuan Agricultural University, Chengdu, China; 5 Triticeae Research Institute, Sichuan Agricultural University, Chengdu, China

**Keywords:** *Aegilops tauschii*, selenium, zinc, QTL, molecular markers, metabolism

## Abstract

Crop selenium and zinc biofortification relies heavily on bioaccumulation efficiency, whereas the genetic and molecular mechanisms mediating selenium (Se) and zinc (Zn) accumulation in Aegilops tauschii are still poorly understood. In this study, quantitative trait locus (QTL) mapping was carried out for selenium and zinc content in a recombinant inbred line (RIL) population derived from a cross between AS2407 (Aegilops tauschii subsp. strangulata) and AS65 (*Aegilops tauschii* subsp. *tauschii*). Two major QTLs, *QSe-1D* and *QZn-2D*, controlling Se and Zn content were identified on chromosomes 1D and 2D, respectively. *QSe-1D* was mapped to a 0.5 cM interval with 11.94% phenotypic variance explained (PVE), while *QZn-2D* was located within a 1.1 cM interval and explained 13.49% PVE. Through an integrated analysis of functional annotation, expression data, and conserved domain variations, we identified three putative genes linked to indirect selenium accumulation mechanisms and three candidates involved in zinc metabolism as the most promising targets. SSR markers (SSR-1D7 and SSR-2D2) closely linked to these two QTLs were developed, and their predictive efficiency was validated in a natural population consisting of 126 *Ae. tauschii* accessions. These findings contribute to deciphering the genetic basis of Se and Zn accumulation, offering theoretical references for marker-assisted selection in biofortification breeding.

## 1 Introduction

Microelement malnutrition is a worldwide human health problem. Approximately 3 billion people are affected by deficiencies in iron (Fe), zinc (Zn) and selenium (Se) (Brundtland, 2002; Welch and Graham, 2004; White and Broadley, 2009; Wu et al., 2018). Breeding programs in recent decades have focused mainly on improving grain production and rarely on enhancing the nutrient content of crops. Consequently, the edible parts of many major staple crops are not optimized with micronutrients, which leads to global micronutrient deficiency, particularly in developing countries (Brundtland, 2002; Welch and Graham, 2004; White and Broadley, 2009; Pu et al., 2014). Breeding staple crops with high micronutrient content is an effective way to overcome nutritional deficiency (Delfini et al., 2021). Se, recognized as an essential trace element for humans and animals, consists of the key component of more than 25 mammalian selenoenzymes or selenoproteins with important biological functions (Rayman, 2002). The active role of Se as an antioxidant, thyroid hormone and general immune function regulator has been highlighted since the essentiality of Se as a nutrient for humans and animals was first established in the 1950s by Schwarz and Foltz (Rayman, 2000; Schwarz and Foltz, 2002). Zn is an essential micronutrient for plants, humans and animals. Zn deficiency is a well-documented world health problem (Rehman et al., 2018). Zn plays a role in more than 300 enzymes and is a constituent in thousands of proteins, including transcription factors (Hänsch and Mendel, 2009). Zn status in plants is directly correlated with plant growth, crop yield, and product nutritional quality (Cakmak, 2008; Sadeghzadeh and Rengel, 2011).

Humans mainly intake Se and Zn from Se- and Zn-rich diets. The absorption and bioaccumulation capacities of plants are crucial determinants of Se and Zn levels in food supplies. Common wheat (*Triticum aestivum* L., genome AABBDD), one of the main staple crops worldwide that supplies a major energy source and nutrients for a large number of people in the world, is an allohexaploid that originated from hybridization between cultivated tetraploid wheat (*Triticum turgidum*, AABB) and the diploid *Aegilops tauschii* (*Ae. tauschii*, DD) (Mcfadden and Sears, 1946; Evans and Wardlaw, 1996). Since only limited tetraploid and diploid genotypes were involved in its origination, common wheat has reduced genetic diversity compared with its donor species. The genetic variation of bread wheat has been further narrowed by subsequent domestication and modern breeding (Shewry, 2009). Wild wheat relatives exhibit abundant genetic variation. Previous studies showed that *Ae. tauschii* exhibited higher and more abundant genetic variations in selenium concentrations than bread wheat (Cakmak, 2008; Wu et al., 2018; Zhang and Chu, 2022; Somagattu et al., 2024). *Ae. tauschii* is therefore a promising source for improving selenium and zinc accumulation of common wheat (Somagattu et al., 2024). Understanding the genetic control of Se and Zn in *Ae. tauschii* is thus essential for improvement common wheat Se and Zn content by biotechnological as well as synthetic hexaploid wheat approaches.

The accumulation of Se and Zn in crops is a complex physiological process, involving uptake, transport, translocation and accumulation, and any link in these processes plays a crucial role in the enrichment of selenium and zinc in grains (Gupta et al., 2016; Hossain et al., 2021; Zhang and Chu, 2022; Somagattu et al., 2024). Absorption and translocation serve as core physiological processes governing selenium and zinc accumulation in cereal crops. Shoots (stems and leaves) serve as the primary source-sink organs responsible for Se uptake, assimilation and translocation. After being absorbed by roots, Se and Zn are first accumulated in aboveground tissues, and then transported to reproductive organs such as grains via phloem pathways (Hossain et al., 2021; Neeraja and Suman, 2023). Some studies have verified that Se and Zn contents in shoot tissues are closely related to those in cereal grains (Zhang et al., 2006; Hussain et al., 2016; Zhang and Chu, 2022). It has been documented that elevated Se concentration in rice seedling leaves facilitates more selenomethionine (SeMet) release through protein degradation during senescence, which accelerates SeMet translocation to grains and ultimately increases grain Se content (Zhang and Chu, 2022). Wheat grain Se concentration presents an extremely significant positive correlation with plant Se accumulation under both Se fertilization and non-fertilization conditions (Liu, 2016).

In our previous study, different *Ae. tauschii* accessions were evaluated for their Se and Zn concentrations (Zhao et al., 2017). Among these accessions, *Ae. tauschii* accession AS2407 exhibited a high capacity for Se and Zn accumulation, whereas AS65 showed the opposite pattern. To clarify the genetic mechanisms underlying Se and Zn accumulation in *Ae. tauschii*, we employed RAD-seq to identify SNPs and perform high-throughput genotyping of a RIL population derived from a cross between AS2407 and AS65. Under hydroponic culture conditions, the concentrations of Se and Zn in shoot tissues were quantified using ICP-MS. By combining phenotypic and genotypic data, genetic loci governing Se and Zn accumulation were identified, and closely linked molecular markers for these target genomic regions were developed and validated.

## 2 Materials and methods

### 2.1 Plant materials

A previous study has revealed a significantly higher selenium concentration in the seedlings of AS2407 (*Ae. tauschii* ssp. *strangulata*) than in those of AS65 (*Ae. tauschii* ssp. *tauschii*) (Zhao et al., 2017). A mapping population consisting of 95 F_7_ recombinant inbred lines (RILs) derived from a cross between AS2407 and AS65 was used in the current study for genetic mapping. The natural population of *Ae. tauschii* used to validate molecular markers comprised 126 accessions from 12 countries (**Supplementary Table 3**).

### 2.2 Experimental design and implementation

The seeds of AS2407, AS65, the 95 RILs and 126 natural accessions were soaked in double-distilled water (ddH_2_O), placed into a refrigerator at 4°C for 24 h for dormancy breaking, and then germinated in sterilised Petri dishes with moist filter paper at a constant temperature (25°C) in darkness. At the three-leaf stage, the seedlings were transplanted into 72-well seedling hole trays (530 mm × 270 mm × 40 mm) containing a quarter of Hoagland’s nutrient solution under daily conditions in a greenhouse (day: 25°C, 16 h; dark: 20°C, 8 h) for growth. During the seedling growth period, the humidity was adjusted to approximately 60%. Zinc is supplied by zinc sulphate (ZnSO_4_), which is one of the components of Hoagland’s nutrient solution. After two weeks, half of the seedlings were treated with sodium selenate (Na_2_SeO_4_) to a final concentration of 10 μM. This concentration was selected based on a previous transcriptome study on *Ae. tauschii* which demonstrated that 10 μM Na_2_SeO_4_ effectively induces Se metabolism responses in this species (Wu et al., 2019). A randomized block design with three replicates was used in the study, and each replicate comprises three plants. Two weeks later, the aerial parts of all samples were collected for element measurement and genomic DNA extraction. All lines contain three biological replicates. The solution was replaced by a fresh solution every 2 days during plant growth to ensure a constant concentration and pH value.

### 2.3 Element measurement

The aerial parts of seedlings were harvested, dried at 80°C to constant weight, and ground to a fine powder. Approximately 0.50 g of sample was digested in Teflon vessels with 5.0 mL of concentrated HNO_3_ (65%, trace metal grade) and 1.0 mL of H_2_O_2_ (30%, trace metal grade) using a microwave digestion system (temperature program: 120°C → 150°C → 170°C → 190°C, total 50 min). After cooling, the digestates were diluted to 50 mL with ultrapure water and allowed to stand overnight. Element concentrations were determined by inductively coupled plasma mass spectrometry (ICP-MS, Beijing Laixi Testing Technology Co., Ltd.), with calibration curves established using mixed standard solutions prepared from Se and Zn stock solutions. All analyses followed the method described by Ammann (2007).

### 2.4 Statistical analysis

Data organization and basic statistical analysis were performed using Microsoft Excel 2019 (Microsoft Corp., Redmond, WA, USA). Normality tests were conducted in OriginPro 2018 (OriginLab Corp., Northampton, MA, USA), and analysis of variance (ANOVA) was performed using IBM SPSS Statistics 18.0 (IBM Corp., Armonk, NY, USA). Broad-sense heritability (*H²*) was estimated using a mixed linear model: *Y_ij_=μ+G_i_+e_ij_*, where *Y_ij_* is the phenotypic value, *μ* is the overall mean, *G_i_* is the random effect of genotype (ID), and *e_ij_* is the residual error. Variance components were estimated via restricted maximum likelihood (REML) using the lme4 package (version 1.1-35) (Bates et al., 2015) in R software. Heritability was calculated as. H² was calculated as *H*^2^=*V_G_*/(*V_G_*+*V_E_*), where *V_G_* is the genetic variance and *V_E_* is the error variance.

### 2.5 RAD-seq analysis

The plant genomic DNA kit (TIANGEN Biotech, Beijing, China) was used to extract DNA from the parents and all RILs. A total of 1 μL of DNA solution was used to identify the DNA quality in 1% agarose gels by electrophoresis, and the DNA concentration was measured with a Nano Drop 2000C spectrophotometer (Thermo Scientific, Wilmington, DE, USA). Qualified genomic DNA was used to construct a RAD-seq library (NEB Next^®^ MLtra™ DNA Library Prep Kit, NEB, USA) and sequenced on an Illumina NovaSeq 6000 (Illumina, San Diego, CA, USA) using 150 bp paired-end reads.

### 2.6 Construction of a high-density genetic map

Raw data were filtered using FASTP v18.0 (Chen et al., 2018) under the following rules: (1) reads with >10% unknown bases (N) were discarded; (2) reads with over 50% bases showing a Phred quality score ≤ 20 were removed; (3) adapter-contaminated reads were eliminated. Clean reads were aligned to the reference genome Aet_T093_v1.0 using Burrows-Wheeler Aligner (BWA) to identify single nucleotide polymorphisms (SNPs). Variant calling across all samples was implemented with the UnifiedGenotyper tool in the Genome Analysis Toolkit (GATK). We retained markers with homozygous polymorphisms and sequencing depth > 4×. Markers exhibiting a missing rate > 10%, heterozygosity > 75% and severe segregation distortion (P < 0.001) were excluded. The remaining markers were applied for genetic map construction using the OrderMarkers2 module of LEP-MAP3 (identical limit=0.01, min error=0.001, sex averaged=0, informative mask=123) (Rastas, 2017).

### 2.7 QTL analysis and candidate gene screening

QTL analysis was performed with QTL IciMapping 4.1. QTLs for selenium and zinc concentrations were detected in the biparental population (BIP) via inclusive composite interval mapping (ICIM). The parameters were set as: scan speed = 1.00 cM and stepwise regression threshold = 0.001. The LOD significance thresholds were determined by 1,000 permutation tests at a Type I error rate of 0.05. A fixed LOD threshold of 2.0 was also applied to cross-validate the results. Genetic maps were visualized with MapChart 2.2. QTLs with phenotypic variation explained (PVE) > 10% were designated as major-effect QTLs, following the widely adopted criterion in plant QTL mapping studies (Wang H. et al., 2021; Li et al., 2025).

In addition, to evaluate potential epistatic interactions between the QTLs on chromosomes 2D and 3D, two-way ANOVA was performed with Zn concentration as the dependent variable and genotypes at the two peak markers as fixed factors, including their interaction term. Individuals with missing genotype data were excluded, and P < 0.05 was considered significant for epistasis.

Gene annotation and enrichment analysis were carried out using KOBAS-intelligence (http://bioinfo.org/kobas) against the genome assembly (https://db.cngb.org/search/assembly/CNA0019289). GO and KEGG enrichment analyses were applied to characterize gene functions. Genes participating in zinc and selenoprotein metabolism were chosen as candidate genes for further investigation.

Primers targeting annotated genes were designed via Primer-BLAST (https://www.ncbi.nlm.nih.gov/tools/primer-blast/index.cgi?LINK_LOC=BlastHome) and synthesised by Azenta Life Sciences (GENEWIZ). After amplification and purification (**Supplementary Table 1**), candidate gene fragments were subjected to Sanger sequencing at GENEWIZ. The 50 μL PCR mixture consisted of 25 μL PrimeSTAR mix, 2 μL forward primer, 2 μL reverse primer, 2 μL template DNA and 19 μL ddH_2_O. Align-x was used for sequence alignment.For candidate gene selection, the LOD-1.5 confidence intervals for each QTL were calculated using R/qtl. Putative candidate genes were then identified within these confidence intervals based on functional annotation and physical proximity to the QTL peak markers, with priority given to genes functionally relevant to Se and Zn metabolism. Gene models were retrieved from the Aet_T093_v1.0 reference genome.

### 2.8 Development and validation of markers

SSR markers were mined in QTL regions of chromosomes 1D and 2D via Krait v1.5.1 (ultra-fast SSR search module) and Primer3 (**Supplementary Table 2**). We screened perfect SSR motifs, including mono- to hexanucleotides, with the minimum repeat numbers set to 12, 6, 5, 4, 4 and 4 in sequence (Du et al., 2018). SSR-PCR products were separated on a 10% non-denaturing polyacrylamide gel (PAGE). A 55 mL gel solution was prepared by mixing 30% acrylamide (18 mL), 5× TBE buffer (11 mL), ddH_2_O (25 mL), TEMED (36 µL), and 10% APS (385 µL), with APS and TEMED added last immediately before casting the gel. PCR products (5 µL) were loaded into each well and electrophoresed in 1× TBE buffer at a constant voltage of 120 V. After electrophoresis, the gel was visualized using a silver staining kit (Solarbio, G7210) for nucleic acid PAGE according to the manufacturer’s protocol.

## 3 Results

### 3.1 Statistical analysis of the concentrations of zinc and selenium

**Figure 1:**
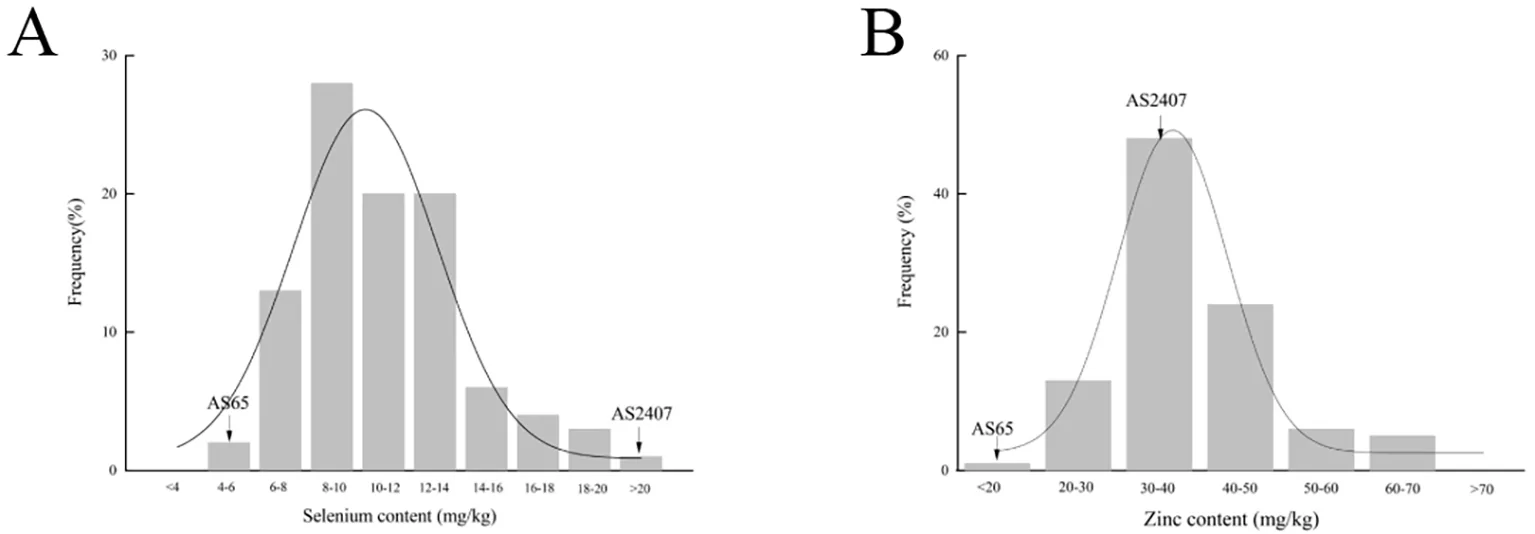
Distribution of selenium **(A)** and zinc **(B)** concentrations observed in shoot tissues of the RIL population.

The concentrations of selenium and zinc in the two parents and 95 RILs were measured by ICP-MS. The results showed that: (1) AS2407 exhibited 6- and 2-fold higher concentrations of Se and Zn than AS65 in hydroponically cultivated seedlings, respectively (**Table 1**). Significant differences in the concentrations of zinc and selenium between the two parents were detected (p<0.05). (2) The average content of selenium and zinc in the RIL population was 10.89 mg/kg and 39.03 mg/kg, and the coefficients of variation were 28% and 25%, respectively. Selenium content in 70% of the RILs ranged from 8 mg/kg to 14 mg/kg (**Table 1**; **Figure 1A**). Up to 68% of the RILs showed higher zinc concentrations than AS2407 (**Table 1**; **Figure 1B**). The broad-sense heritability for Se and Zn concentration was estimated to be 0.739 and 0.579, respectively. Correlation analysis revealed a significant positive correlation between Se and Zn concentrations in the shoots of *Ae. tauschii* accessions (Pearson’s r = 0.24, P < 0.01; **Table 1**).

**Table 1:** Descriptive statistics for selenium and zinc content of parents and a RIL population.

Content(mg/kg)	Parents		RILs population							
Trait	P1	P2	Mean	SD	CV (%)	SK.	Ku.	*H^2^*	S-W P	r
Se	31.05	5.36	10.89	3.07	28	1.12	0.93	0.74	< 0.001	0.24^**^
Zn	33.25	16.68	39.03	9.65	25	1.17	1.01	0.58	< 0.001	

P1: AS2407; P2: AS65; SD: standard deviation; CV: coefficient of variation; SK.: skewness; Ku.: kurtosis; H2: broad-sense heritability; S-W P: P value of Shapiro–Wilk normality test; r: correlation coefficient. ** represents extremely significant correlation at (P<0.01).

### 3.2 Construction of high-density genetic maps and QTL analysis

A total of 4,222,948,582 raw RAD-seq reads were generated from 97 *Ae. tauschii* individuals, with an average of 43,535,552 reads per sample and an actual read length of 142 bp. Based on the haploid genome size of the *Ae. tauschii* reference genome Aet_T093_v1.0 (4.3 Gb), the average raw sequencing coverage was approximately 18.54× per sample. After filtering for high-quality reads (97.11%), the effective sequencing coverage reached 18.00×. A total of 12,944,233 high-quality single nucleotide polymorphisms (SNPs) and indels were identified in the parental lines and the RIL population via RAD-seq analysis. Following filtering of redundant markers, 930,562 polymorphic markers were used to construct a high-density genetic map spanning 884.313 cM (**Table 2**). These markers were distributed across all seven chromosomes, with linkage group lengths ranging from 87.385 cM to 174.752 cM. Chromosome 3D harbored the largest number of polymorphic markers (196,827), whereas chromosome 4D contained the fewest (10,838). The average interval between adjacent markers was only 0.001 cM (**Table 2**). The 930,562 markers were grouped into 1,308 bin markers, with an average genetic distance of 0.680 cM between consecutive bins. The physical length of bin markers ranged from 0.001 kb to 184.735 Mb, with a median length of 218 kb. Overall, 75% of the bin markers were less than 1.6 Mb in length. The number of bins per chromosome varied from 115 (Chr4D) to 276 (Chr7D). The order of almost all SNP markers in the genetic map was consistent with the reference genome in *Ae. tauschii*.

**Table 2:** Characteristics of the high-density linkage map developed using RAD-seq SNP markers.

Chrome	Length (cM)	Marker (num)	Average (cM)	Number of Bin markers	cM per Bin marker
Chr1D	104.221	142,868	0.001	154	0.68
Chr2D	120.537	196,717	0.001	191	0.63
Chr3D	114.748	196,827	0.001	173	0.66
Chr4D	87.385	10,838	0.008	115	0.76
Chr5D	172.130	171,493	0.001	238	0.72
Chr6D	110.540	151,106	0.001	161	0.69
Chr7D	174.752	60,713	0.003	276	0.63
Total	884.313	930,562	0.001	1308	0.68

**Figure 2:**
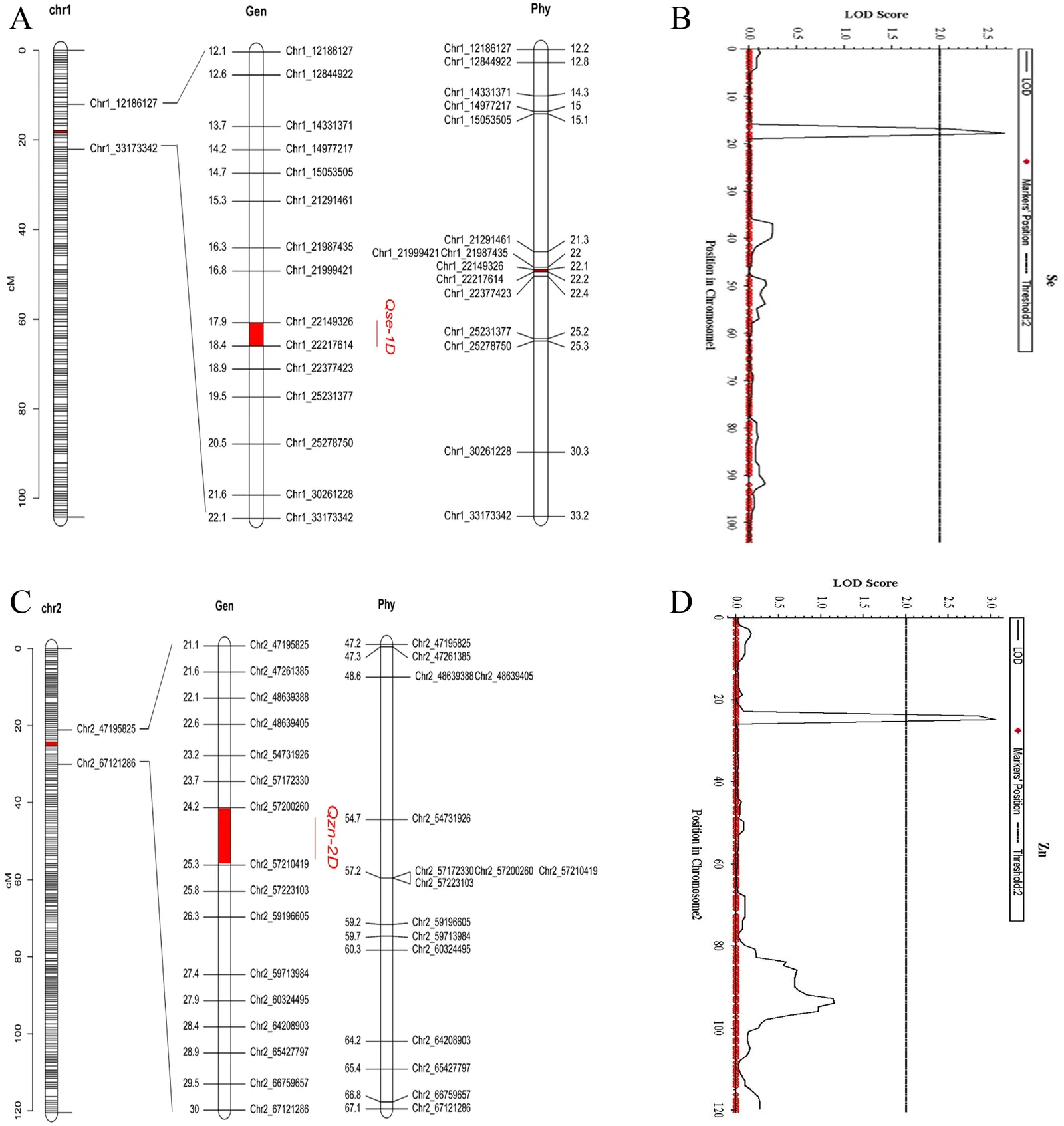
Genetic, physical maps and the LOD value distribution of major QTLs. **(A)** *s*hows the full-length 1D chromosome as well as the genetic and physical maps of major QTL loci (*QSe-1D*) associated with selenium content. The red bar represents the genetic intervals **(B)** shows the the LOD value distribution of *QSe-1D*. **(C)** *s*hows the full-length 2D chromosome as well as the genetic and physical maps of major QTL loci (*QZn-2D*) associated with zinc content. The red bar represents the genetic intervals **(D)** shows the the LOD value distribution of *QZn-2D*.

**Figure 3:**
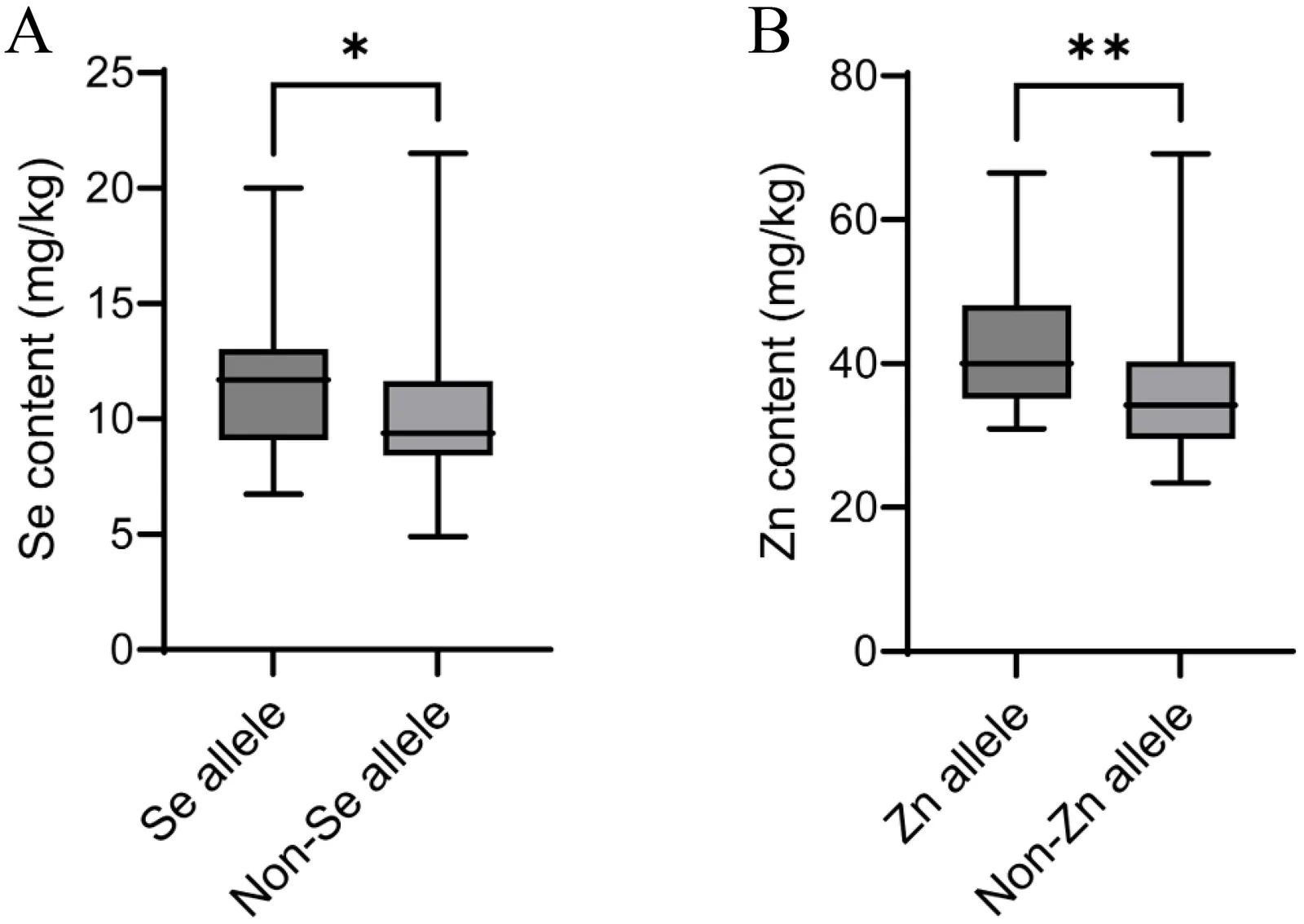
The significant differences of Se and Zn content between RILs with and without favorable alleles. **(A)** Se content differed significantly between 42 lines with Se allele and 45 lines without Se allele (*: P < 0.05). **(B)** Zn content showed extremely significant differences between 35 lines with Zn allele and 44 lines without Zn allele (**: P < 0.01).

The BIP function of IciMapping with the ICIM-ADD module was used to analyze the loci related to selenium and zinc concentrations of the RIL population. The LOD significance thresholds were determined by 1,000 permutation tests at a Type I error rate of 0.05. Two major QTLs, *QSe-1D* and *QZn-2D*, were identified: (**Figure 2**). *QSe-1D* was located on chromosome 1D in the interval of Chr1_22149326 and Chr1_22217614 at 70 kb, within a 0.5 cM genetic distance from 17.9 cM to 18.4 cM (**Table 3**). The phenotypic variance explained (PVE) by *QSe-1D* was 11.94% (**Table 3**). *QZn-2D* was located at 10 kb with a flanking region between Chr2_57200260 and Chr2_57210419 on chromosome 2D, and the genetic distance was 1.1 cM from 24.2 cM to 25.3 cM (**Table 3**). The PVE by the *QZn-2D* was 13.49% (**Table 3**). In the RIL population, the lines carrying the *QSe-1D* and *QZn-2D* alleles had significantly higher (p<0.05, p<0.01) Se (27.56%) (**Figure 3A**) and Zn (17.11%) (**Figure 3B**) concentrations than those lines without *QSe-1D* and QZn-2D, respectively. Additionally, a minor QTL (*QZn-3D*) associated with Zn concentration was identified on chromosome 3D (LOD = 2.18, PVE = 8.52%) using the fixed LOD threshold of 2.0, though it did not reach significance under the 1000 permutation test (**Supplementary Figure 1**). *QZn-3D* was flanked by markers Chr3_482421352 and Chr3_484357914, with a PVE of 8.52% (**Table 3**). Based on additive effect estimates, the favorable alleles for *QSe-1D* and *QZn-2D* were contributed by the parent AS2407. In contrast, the favorable allele for *QZn-3D* originated from AS65.

**Table 3:** QTLs were detected for Se and Zn content in RILs.

Trait	QTL	Chr	Peak (cM)	Flanking markers	LOD	Add	PVE%	Physical position (Mb)
Se	*QSe-1D*	1D	18.00	Chr1_22149326- Chr1_22217614	2.68	1.28	11.94	22.15-22.22
Zn	*QZn-2D*	2D	25.00	Chr2_57200260- Chr2_57210419	3.05	3.61	13.49	57.20-57.21
	*QZn-3D*	3D	69.00	Chr3_482421352- Chr3_484357914	2.18	2.88	8.52	482.42-484.36

Chr, chrome; LOD, logarithm of odds; Add, the additive effect; positive values indicate that the alleles controlling Se and Zn content was derived from AS2407; while negative values represented those derived from AS65. PVE, phenotypic variation explained. PVE exceeding 10% were defined as major QTLs. Physical positions shown are ICIMapping peak intervals.

Notably, the highest Zn concentration was observed in lines carrying favorable alleles at both *QZn-2D* and *QZn-3D* (40.56 ± 12.43 mg kg^−^¹), whereas lines with either single favorable allele showed intermediate levels (38.90 and 38.79 mg kg^−^¹; **Table 4**). This pattern supports the additive combination of complementarily distributed favorable alleles from the two parents.

**Table 4:** Pyramiding effects of *QZn-2D* and *QZn-3D* in RILs.

*QZn-2D*	*QZn-3D*	N	Zn (mg/kg)
–	–	21	37.83 ± 9.46
+	–	14	38.90 ± 10.79
–	+	19	38.79 ± 9.38
+	+	23	40.56 ± 12.43**

“-” and “+” represent absence and presence of the favorable allele at each QTL, respectively. N = the number of RILs in each group. Values of Zn concentration are presented as mean ± standard deviation. “_**_” represents significant at P<0.01 relative to the control group.

### 3.3 Potential candidate genes of *QSe-1D* and *QZn-2D*

**Figure 4:**
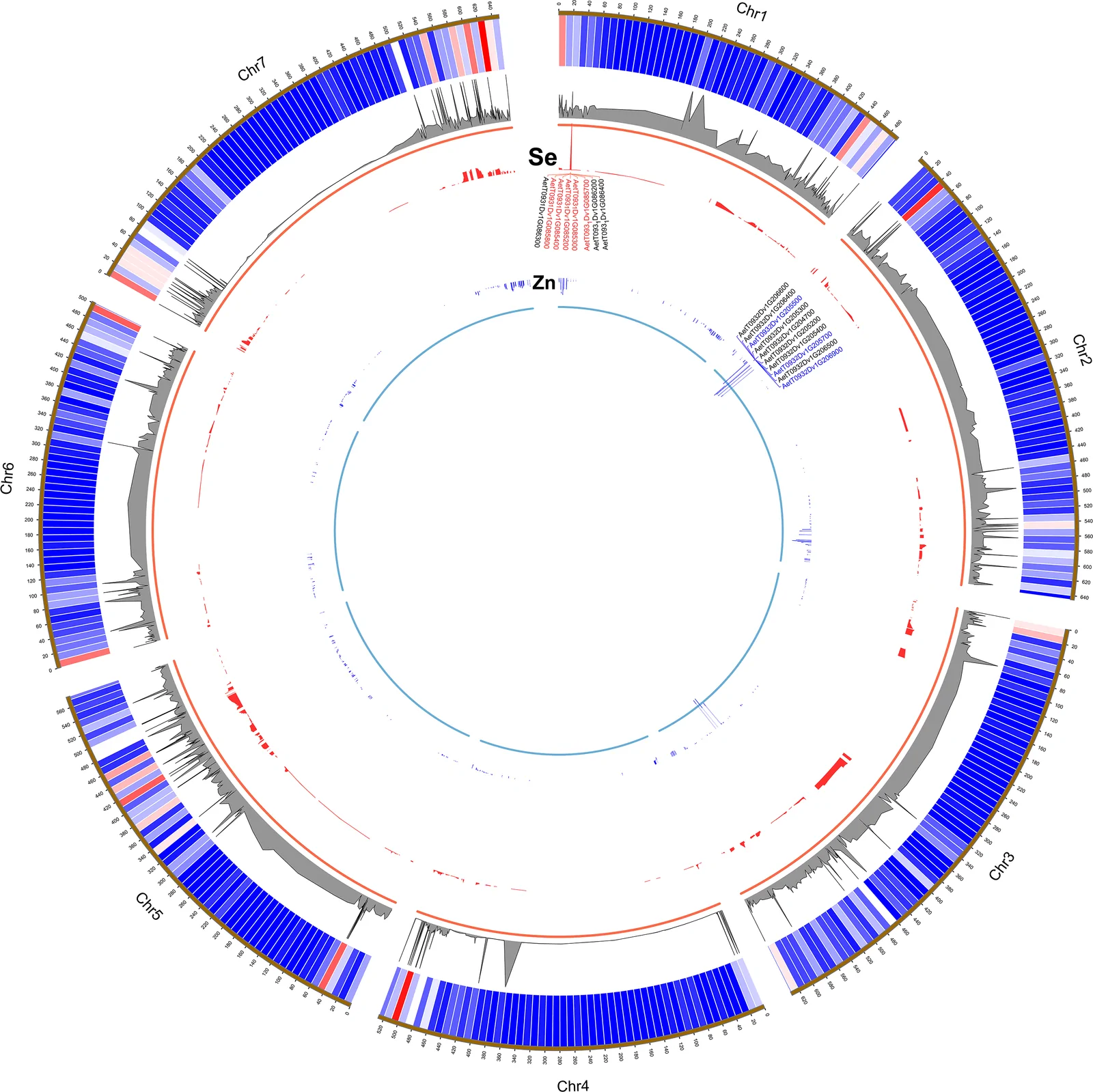
Genome circle map of major QTLs. From outer to inner circles: 1) Molecular markers and an SNP density heat map (blue to red gradient, with a complementary gray density profile); 2) LOD score profiles for Se (red) and Zn (blue) QTLs, with solid circles marking the significance thresholds; 3) Annotations of candidate genes identified at QTL peaks (Se-related in red, Zn-related in blue).

**Figure 5:**
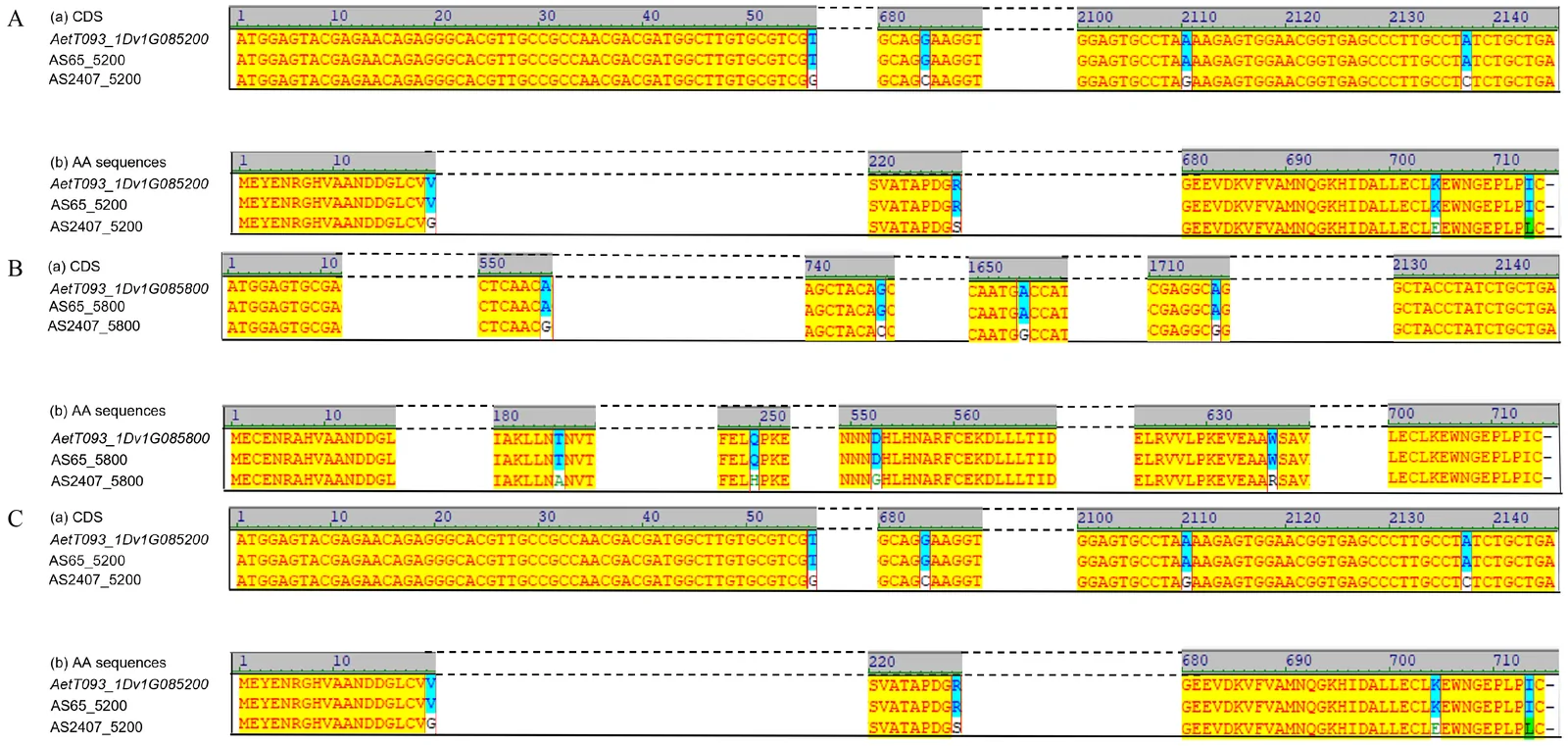
Non-synonymous Mutations in Selenium-Associated Genes. Non-synonymous mutations identified in genes *AetT093_1Dv1G085200* **(A)**, *AetT093_1Dv1G085800* **(B)**, and *AetT093_1Dv1G086500* **(C)** are presented. For each gene, the coding DNA sequence (CDS) and the corresponding amino acid sequence are shown in panels **(a, b)**, respectively. Nucleotide variations (SNPs) specific to each gene in comparison to AS65 are highlighted in blue. Red indicators mark the precise locations of the mutations in both the DNA and amino acid sequences. Standard single-letter amino acid codes are used: V (Valine), R (Arginine), K (Lysine), I (Isoleucine), G (Glycine), S (Serine), E (Glutamic acid), L (Leucine), T (Threonine), Q (Glutamine), D (Aspartic acid), W (Tryptophan), H (Histidine).

We identified three Potential candidate genes that participate in a co-regulated phenylpropanoid/flavonoid pathway, which may indirectly influence selenium accumulation through shared stress/redox regulation. (**Table 5**; **Figure 4**). *AetT093_1Dv1G085200* and *AetT093_1Dv1G08580* encode phenylalanine ammonia-lyase (PAL), *AetT093_1Dv1G086500* encoded chalcone synthase 2-like (CHS2) (**Table 5**, **Figure 4**), which were key enzymes in Biosynthesis of secondary metabolites. we designed corresponding primers to explore the differences of selenium-related genes in parents. Compared with Aet_T093_v1.0 genome, *AetT093_1Dv1G085200*, *AetT093_1Dv1G085800*, *AetT093_1Dv1G086500* had SNPs variants in AS2407 genes and had not variations in AS65. *AetT093_1Dv1G085200* had 12 SNPs variants in Coding DNA Sequence (CDS), among which 4 non-synonymous mutations and 8 synonymous mutations (**Table 6**; **Figure 5A**). *AetT093_1Dv1G085800* had 13 SNPs variants in CDS of AS2407 (**Table 6**; **Figure 5B**). There were 4 non-synonymous mutation and 9 synonymous mutation *AetT093_1Dv1G086500* had 5 SNPs variants in CDS of AS2407, which included 2 non-synonymous mutation and 3 synonymous mutation (**Table 6**; **Figure 5C**). In addition, indels were found in the upstream of *AetT093_1Dv1G085200* CDS (including 24bp insertion and1bp deletion) in AS2407 (**Table 6**).

**Table 5:** Putative candidate genes for QSe-1D and QZn-2D with their physical positions and functional annotations.

Trait	Gene ID	Gene Annotation information	Physical/Mb
Se	*AetT093_1Dv1G085200*	PAL	22.150-22.153
	*AetT093_1Dv1G085800*	PAL	22.217-22.220
	*AetT093_1Dv1G086500*	CHS2	22.356-22.357
Zn	*AetT093_2Dv1G206900*	zinc finger CCCH domain	58.178-58.182
	*AetT093_2Dv1G205500*	NAM-B1-like	57.544-57.547
	*AetT093_2Dv1G205700*	NAM-B1 protein ATAF2-like	57.692-57.695

Gene IDs and Physical positions are based on the Aet_T093_v1.0 (CNA0019289) reference genome annotation. PAL, phenylalanine ammonia-lyase; CHS2, chalcone synthase 2-like; NAM-B1, NAC transcription factor; CCCH, tandem CCCH zinc finger domain.

**Table 6:** Non-synonymous mutations identified in the putative candidate genes of Se metabolism between parental lines AS2407 and AS65.

Putative functional genes	Primers	SNP			AA		
		AetT093	AS2407	AS65	AS2407	AS65	Loci
*AetT093_1Dv1G085200*	chr1D_ 085200	T	G	T	G	V	19
		G	C	G	S	R	228
		A	G	A	E	K	704
		A	C	A	L	I	713
*AetT093_1Dv1G085800*	chr1D_ 085800	G	A	G	A	T	186
		G	C	G	H	Q	249
		A	G	A	G	D	552
		T	C	T	R	W	636
*AetT093_1Dv1G086500*	chr1D_ 086500	A	G	A	G	D	186
		G	A	G	E	G	285

Comparative analysis with the reference genome (Aet_T093_v1.0) identified SNPs within three candidate gene amplicons (chr1D_085200, chr1D_085800 and chr1D_086500) in parent AS2407, but not in AS65. These CDS variants in AS2407 resulted in both non-synonymous changes. “AA” is the abbreviation for amino acid. This column presents the corresponding amino acid substitutions caused by SNP sequence variations. The SNP loci and corresponding amino acid positions (using standard abbreviations: V, R, K, I, G, S, E, L, T, Q, D, W, H) are listed.

we conducted multiple sequence alignment of the PLN02457 conserved catalytic domain of PAL (*AetT093_1Dv1G085200* and *AetT093_1Dv1G085800*) using the NCBI Conserved Domain Database (CDD). Alignment against the reference PAL protein (GI: 15228074) defined the boundaries of the PLN02457 functional domain as amino acid residues 176 to 616. A C-to-G transversion causing an arginine-to-serine substitution at residue 228 within this catalytic core region. Cross-species alignment of representative terrestrial plant PAL homologs (GI: 168006654, 168007404, 168018884, 168067524) demonstrated that residue 186 is fully conserved as threonine. In *AetT093_1Dv1G085800*, a non-synonymous SNP mutates this invariant threonine to alanine. For *AetT093_1Dv1G086500* (CHS2), non-synonymous mutations were detected within its conserved functional domains, consistent with its role as a structurally conserved enzyme in the flavonoid biosynthesis pathway.

Three genes were found to be associated with zinc accumulation (**Table 5**; **Figure 4**). *AetT093_2Dv1G206900* encoded a series of CCCH-type zinc finger proteins. *AetT093_2Dv1G205500* and *AetT093_2Dv1G205700* encoded NAM-B and ATAF2 protein belong to the NAC-type transcription factor family (**Table 5**). Indels were detected in the downstream of *AetT093_2Dv1G205500* CDS (including 2bp insertion and1bp deletion) in AS2407 (**Table 6**). No variants were detected in CDS of these genes, although sequence polymorphisms were identified in the upstream regulatory region of *AetT093_2Dv1G205500*.

### 3.4 Development of microsatellite markers and their validation

**Figure 6:**
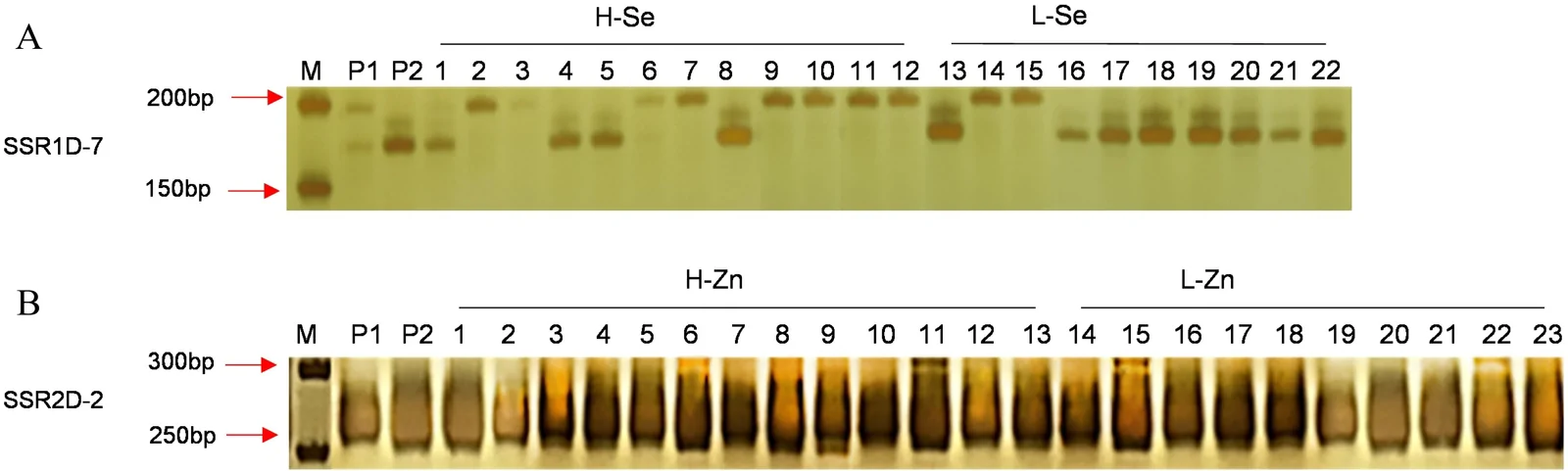
Genotyping of extreme Se **(A)** and Zn **(B)** accumulation groups with SSR markers. The high (H-Se/Zn) and low (L-Se/Zn) lines from a natural population were analyzed using SSR1D-7 (Se) and SSR2D-2 (Zn) Where, M: 500 bp ladder; P1, P2: AS2407and AS65; H-Se/Zn and L-Se/Zn: the extreme high Se/Zn group and low Se/Zn group.

A total of 9 and 5 SSR markers were successfully developed in the region of *QSe-1D* and *QZn-2D*, respectively (**Supplementary Table 1**). Markers (SSR-1D7 and SSR-2D2) were showed parental polymorphism, which were used to validate. The results showed that SSR-1D7 and SSR-2D2 can classify the genotypes of the 126 accessions of *Ae. tauschii* into two classes, respectively (**Supplementary Figure 2**). Among these accessions, for SSR-1D7 and SSR-2D2, 41 and 60 *Ae. tauschii* accessions exhibited the same genotype as AS2407 (genotype: H-Se/Zn). On the other hand, 82 and 52 *Ae. tauschii* accessions exhibited the same genotype as AS65 (genotype: L-Se/Zn). 3 and 14 *Ae. tauschii* accessions did not amplify bands by SSR-1D7 and SSR-2D2, respectively (**Supplementary Table 2**). Based on the continuous distribution analysis of selenium and zinc contents in the natural population, 12 high Se content (>22 mg/kg) and 10 low Se content (<11 mg/kg) lines were screened, and 13 high Zn content (>37 mg/kg) and 10 low Zn content (<22 mg/kg) lines were selected from extreme phenotypic lines (**Table 7**; **Figure 6**). The markers SSR-1D7 and SSR-2D2 were validated in extreme high and low phenotypic lines. Among the Se and Zn extreme lines (22 and 23 lines, respectively), 16 and 14 lines showed consistent phenotypes and genotypes. The prediction efficiency of the SSR-1D7 and SSR-2D2 were 72.73% and 60.87%, respectively (**Figure 6**).

**Table 7:** Phenotypes and genotypes of extreme Se- and Zn-accumulation lines in natural population.

Traits	Serial number	Accession	Phenotype(mg/kg)	Genotype
H-Se	1	AS4	22.12	L
	2	AS114	22.68	H
	3	AS43	23.11	H
	4	AS73	23.32	L
	5	AS42	23.41	L
	6	AS83	23.57	H
	7	AS76	25.53	H
	8	AS68	26.24	L
	9	AS81	27.16	H
	10	AS124	28.30	H
	11	AS61	30.14	H
	12	AS27	33.50	H
L-Se	13	AS80	8.23	L
	14	AS78	8.33	H
	15	AS79	8.80	H
	16	AS32	8.92	L
	17	AS56	9.24	L
	18	AS74	9.30	L
	19	AS24	9.30	L
	20	AS28	10.13	L
	21	AS123	10.24	L
	22	AS70	10.75	L
H-Zn	1	AS18	37.04	L
	2	AS45	37.11	H
	3	AS119	37.41	H
	4	AS34	37.50	H
	5	AS26	37.65	H
	6	AS85	37.90	L
	7	AS100	38.04	H
	8	AS117	38.30	L
	9	AS46	38.66	H
	10	AS110	38.78	H
	11	AS8	40.53	L
	12	AS125	41.74	H
	13	AS113	50.26	H
L- Zn	14	AS63	18.02	H
	15	AS47	18.78	L
	16	AS123	19.57	H
	17	AS33	20.83	H
	18	AS30	21.77	H
	19	AS51	21.26	H
	20	AS1	21.32	L
	21	AS7	21.32	L
	22	AS71	21.39	L
	23	AS99	21.83	L

H-Se/Zn and L-Se/Zn: the extreme high Se/Zn group and low Se/Zn group. H and L represent the AS2407 homozygous allele and the AS65 homozygous allele, respectively.

## 4 Discussion

As the second largest food crop in the world, Se and Zn contents in wheat are important factors affecting human health (Li et al., 2022; Li et al., 2024). Breeding Se- and Zn-enriched wheat varieties is an ideal strategy for improving nutrition in Se- and Zn-deficient regions. Common wheat exhibits narrow genotypic variation in Se concentrations compared with its diploid D genome donor *Ae. tauschii*, which exhibited a great advantage of accumulating selenium (Lyons et al., 2005; Zhao et al., 2017). The higher micronutrient contents of wild species have been often attributed to their small sizes and lower harvest indexes (Calderini and Ortiz-Monasterio, 2003; Oury et al., 2006). *Ae. tauschii* is the typical small sizes and lower harvest indexes plant. The selenium accumulation efficiency and tolerance of plants are important determinants of Se biofortification efficiency. Cereal crops (wheat, rice and barley) accumulate high levels of selenium and zinc in shoot tissues, which are subsequently remobilized to grains during grain filling (Hegelund et al., 2012; Zhang and Chu, 2022; Kong et al., 2024). Thus, total biomass Se- or Zn-enriched *Ae. tauschii* genetic resources would be highly valuable for understanding the genetic basis of Se and Zn accumulation and applying to wheat nutritional quality improvement. In the study, we focused on total biomass Se and Zn-enriched *Ae. tauschii* accession AS2407 and revealed its genetic basis of Se and Zn accumulation.

Many QTLs related to grain Se and Zn content have been detected in common wheat (Shi et al., 2008; Pu et al., 2014; Velu et al., 2017; Wang et al., 2017; Wang W. et al., 2021; Sun et al., 2023; Sun et al., 2024) and synthetic wheat (Pu et al., 2014; Krishnappa et al., 2017; Pu et al., 2018). In addition, QTLs associated with grain Se content were identified in tetraploid wheat (Yan et al., 2018). For grain Zn concentrations, QTLs were reported in the A (Tiwari et al., 2009), B (Peleg et al., 2009) and D (Arora et al., 2019) genome of wheat. Despite a few studies have conducted on the Se and Zn content in wild ancestors of wheat, there is still little research on Se and Zn zinc content in *Ae. tauschii*.

The *Ae. tauschii* possesses abundant genetic diversity, which served as the source of many elite genes in hexaploid wheat, significantly improving micronutrient quality in wheat (Kishii, 2019). As juice powder made from cereal seedlings has become a popular functional food in recent years, for example barley and wheat seedlings, enhancing micronutrient concentration in cereal seedlings is an ideal strategy (Hawkesford and Zhao, 2007; Velu et al., 2014; Zhao et al., 2015). The final content of Se and Zn in plants is controlled by three key steps: uptake, assimilation and translocation (Wu et al., 2019). Plants have the highest Se concentrations in young leaves during seedling growth, and the concentration of selenium decreases as it is transferred from the leaves to the reproductive organs (White, 2016). Furthermore, Previous studies have demonstrated that selenium content in brown rice is positively correlated with the total Se of the whole plant, shoot, and biomass of shoots, and that the total Se transported from roots to shoots is more important than other factors in determining Se accumulation and content in brown rice (Zhang et al., 2006), supporting the relevance of seedling-stage phenotyping for biofortification objectives. To elucidate the molecular mechanisms of Se and Zn accumulation, total biomass was utilized to measure phenotypes, and a hydroponic solution was employed to create consistent nutritional conditions for genotypes in this study.

As far as we know, *QSe-1D* is the first mapped QTL related to Se concentration in *Ae. tauschii.* The additive effect of the loci was positive, indicating that the allele from AS2407 improved Se accumulation. Three putative genes (*AetT093_1Dv1G085200*, *AetT093_1Dv1G085800* and *AetT093_1Dv1G086500*) encoding phenylalanine ammonia-lyase (PAL) and chalcone synthase 2-like (CHS2) were identified as key enzymes in the phenylpropanoid/flavonoid biosynthetic pathway, which is consistent with previous findings (Wu et al., 2018; Pu et al., 2021). These genes are therefore proposed as candidate genes in a co-regulated secondary-metabolism pathway that may influence selenium accumulation through indirect mechanisms, including antioxidant responses to Se-induced oxidative stress and phenylpropanoid-mediated modulation of root architecture (Wang et al., 2016; Guardado-Félix et al., 2017). Furthermore, non-synonymous mutations were detected within its conserved functional domains in *AetT093_1Dv1G085200* and *AetT093_1Dv1G085800*. These suggest that such differences may affect gene function.

Regarding Zn accumulation, *QZn-2D* and *QZn-3D* were identified. *QZn-2D* was designated a major QTL because it accounted for more than 10% of the total phenotypic variation. Potential candidate genes located near *QZn-2D* included *AetT093_2Dv1G205500*, *AetT093_2Dv1G205700* (NAC-type transcription factor) and *AetT093 2Dv1G206900* (a series of CCCH-type zinc finger proteins). The identity of these candidate genes differs from those reported by Arora et al. (2019), suggesting that *QZn-2D* may represent a novel major QTL associated with Zn accumulation in *Ae. tauschii*. *AetT093_2Dv1G205500* and *AetT093_2Dv1G205700* belong to the NAC transcription factor family and contain the NAM domain. The classic NAM-B1 gene (Uauy et al., 2006), located on chromosome 6B, regulates leaf senescence and grain Zn/Fe content in hexaploid wheat. The genes identified here on chromosome 2D of *Ae. tauschii* are paralogs of NAM-B1 in the D genome. Their putative role in Zn accumulation is hypothesized based on functional analogy with NAM-B1, although functional divergence between B- and D-genome paralogs remains to be elucidated. Previous studies (Uauy et al., 2006; Wang et al., 2008; Han et al., 2021) have indicated that NAC transcription factors and CCCH-type zinc finger proteins play pivotal roles in the regulation of Zn accumulation. No variants were detected in CDS of these genes, although sequence polymorphisms were identified in the upstream regulatory region of *AetT093_2Dv1G205500*. To test for potential epistasis between *QZn-2D* and *QZn-3D*, we performed a two-way ANOVA based on the genotypes at the two peak markers. The lack of epistasis between *QZn-2D* and *QZn-3D*, combined with the observation that the highest Zn concentration occurred only when favorable alleles from both loci were stacked, indicates that the additive combination of complementarily distributed parental alleles underlies the transgressive segregation for Zn in this population.

Besides, under Se and Zn treatments, the corresponding candidate genes *AetT093_1Dv1G085200* (FPKM: 9.11 in P1 vs. 0.15 in P2), *AetT093_1Dv1G085800* (FPKM: 3.97 in P1 vs. 1.05 in P2), *AetT093_2Dv1G205500* (FPKM: 18.49 in P1 vs. 2.67 in P2) and *AetT093_2Dv1G205700* (FPKM: 14.32 in P1 vs.1.30 in P2) exhibited significantly higher expression levels in parent AS2407 (P1) compared with AS65 (P2).

Based on QTL confidence intervals, gene annotation, and CDS variation analysis, we propose the following as the most promising candidate genes for Se and Zn accumulation. We acknowledge that the modest population size limits our power to detect small-effect QTLs and may inflate effect estimates (Bennett and Carosone-Link, 2006; King and Long, 2017), necessitating validation in larger populations (Raghavan, 2012). The observed transgressive segregation for both Se and Zn suggests that the two parents contribute complementary favorable alleles (Koide et al., 2019), and reinforces that multiple QTLs with opposing effects are segregating in this population (Xu et al., 1998). Furthermore, minor QTLs and those involved in QTL × environment interactions likely escaped detection in our single-environment experiment, particularly for traits with moderate heritability (David et al., 2023). Thus, the QTLs reported here represent major-effect loci, while minor QTLs and environment-specific effects warrant further investigation.

The QTLs reported herein should be considered as indicative of seedling stage uptake capacity. Validation of these QTLs in field grown plants with grain mineral measurements is warranted in future studies. These putative candidate genes are preliminary and require validation through targeted qRT-PCR across multiple tissues and developmental time points, as well as functional approaches such as virus-induced gene silencing (VIGS) or transgenic analysis in future studies. In addition, the molecular markers SSR-1D7 and SSR-2D2 associated with QTLs for Se and Zn, support their potential utility. The prediction efficiencies of SSR-1D7 and SSR-2D2 were 72.73% and 60.87%, respectively. While SSR-1D7 showed relatively high predictive potential, the modest efficiency of SSR-2D2 suggests that it should be used cautiously in breeding applications and warrants further validation in larger independent populations before any practical implementation. Prediction efficiency can be influenced by QTL effect size, population structure, and marker-QTL linkage disequilibrium; therefore, validation in independent germplasm is essential prior to practical MAS implementation. Nevertheless, both markers provide useful information for understanding the genetic architecture of the target traits and may serve as reference points for fine-mapping efforts.

## Data Availability

The raw sequencing data generated in this study were deposited in the Genome Sequence Archive (GSA) of the National Genomics Data Center (NGDC), Beijing Institute of Genomics, Chinese Academy of Sciences, under accession number CRA046101. The dataset is publicly accessible via https://ngdc.cncb.ac.cn/gsa.
