# Supplementary material for: Identification of novel major quantitative trait loci associated with selenium and zinc accumulation in Aegilops tauschii
Source: Front Plant Sci. 2026 Aug 12;17:1892742. doi: 10.3389/fpls.2026.1892742 (PMC13506878; doi:10.3389/fpls.2026.1892742)
Supplement: Supplementary file 1 [file Table1.docx]

**Supplementary Table S1** Characteristics of the Polymorphic SSR markers

| Primer Name | Primer Sequence (5′-3′) | Tm(°C) | Amplicon size(bp) |
| --- | --- | --- | --- |
| SSR1D-1  SSR1D-2  SSR1D-3  SSR1D-4  SSR1D-5  SSR1D-6  SSR1D-7  SSR1D-8  SSR1D-9  SSR2D-1 SSR2D-2 SSR2D-3  SSR2D-4 | F: TACTTGAGGTATGGGGGCTG R: GGAGAGGTTGGGGAAGACTC  F: GGATCAGAGACCTACCCGTG R: AAAACTGCCACACACGCAC  F: TGGAGAGGAATGGAGGAAGA R: ATCGTGGAAGCAGCCATTAC  F: GTACTCGCCGTGATCCAAAT R: AGGGCAGCAAGATCTAAGCC  F: TTGATGGAGATCCAACAGCA R:GGAGAGTCCTAAAGGGGGAA  F: GAATTGCCTCGTTCTTCAGG R: GTCGCTTTCTGCTTGGATTC  F: CCATGTTTCCTTGACATCCC R: ATGGGTATCGCATGGTCAAT  F: TGGTTGTAGAAGGGGTCGTC R: GAGATGCAAAGAAAGCAGGC  F: ACATATTTCACCTCGCTCGC R: GCCCAAGTTCCTTTTCTTCC  F: GCATAAAGCCCGGAACATAA R: ATGGAACACAGCCAGACTCC  F: CTACCAAGAACTCCCGAACG R: ACCCTCGGGTCTTTCTCATT  F: TGCCCTGGTCAATGTGTAAA R: CATTTGGAGGGAACAGAGGA  F: CGATGATTGATTGATCGTGC R: TCATGACGAAGGGACTACCC | 58  58  60  58  58  60  56  58  59  60  60  58  58 | 177  228  236  260  278  190  188  127  193  249  261  239  255 |
| SSR2D-5 | F: CATAAATCCGGCGATCAAGT R: CTCATCCTGAACCAGCTTCC | 60 | 261 |
