# Supplementary material for: Identification of novel major quantitative trait loci associated with selenium and zinc accumulation in Aegilops tauschii
Source: Front Plant Sci. 2026 Aug 12;17:1892742. doi: 10.3389/fpls.2026.1892742 (PMC13506878; doi:10.3389/fpls.2026.1892742)
Supplement: Supplementary file 2 [file Table2.docx]

**Supplementary Table S2** The information of natural variation in 126 *Ae.tauchii* accessions

| serial number | country/region | Subspecies | Phenotype | | | genotype | |
| --- | --- | --- | --- | --- | --- | --- | --- |
|  |  |  | Se  mg/kg | Zn mg/kg | Se  mg/kg | | Zn  mg/kg |
| AS1 | Iran | *Ae.strangulata* | 11.44 | 21.32 | L | | L |
| AS2 | Turkmenistan | *Ae.tauschii* | 13.22 | 26.33 | H | | L |
| AS3 | N | N | 12.67 | 27.45 | L | | L |
| AS4 | N | N | 22.12 | 30.12 | L | | L |
| AS5 | N | N | 13.78 | 25.56 | L | | H |
| AS6 | N | N | 19.08 | 32.84 | L | | L |
| AS7 | N | N | 14.82 | 21.32 | L | | L |
| AS8 | Pakistan | *Ae.tauschii* | 12.54 | 40.53 | L | | L |
| AS9 | N | N | 17.22 | 28.43 | L | | H |
| AS10 | Turkmenistan | *Ae.tauschii* | 12.64 | 36.01 | L | | L |
| AS11 | Pakistan | *Ae.tauschii* | 13.05 | 23.34 | L | | H |
| AS12 | Pakistan | *Ae.tauschii* | 20.94 | 28.27 | L | | H |
| AS13 | N | N | 15.80 | 26.51 | L | | L |
| AS14 | N | N | 12.90 | 28.84 | L | | H |
| AS15 | N | N | 15.87 | 30.26 | H | | L |
| AS16 | N | *Ae.tauschii* | 14.99 | 25.58 | L | | N |
| AS17 | China | *Ae.tauschii* | 15.04 | 32.73 | L | | N |
| AS18 | Afghanistan | *Ae.tauschii* | 12.87 | 37.04 | H | | L |
| AS19 | N | N | 18.36 | 31.44 | L | | L |
| AS20 | N | N | 16.79 | 30.11 | L | | L |
| AS21 | N | N | 14.33 | 24.59 | L | | H |
| AS22 | Azerbaijan | *Ae.strangulata* | 16.83 | 23.51 | H | | L |
| AS23 | 0 | *Ae.tauschii* | 11.84 | 27.31 | H | | N |
| AS24 | N | N | 9.30 | 33.47 | L | | N |
| AS25 | Iran | *Ae.strangulata* | 16.36 | 26.99 | H | | H |
| AS26 | N | *Ae.tauschii* | 13.89 | 37.65 | H | | H |
| AS27 | Iran | *Ae.strangulata* | 33.50 | 27.33 | H | | L |
| AS28 | N | N | 10.13 | 33.47 | L | | H |
| AS29 | N | N | 20.98 | 26.74 | L | | H |
| AS30 | N | N | 14.82 | 21.77 | L | | H |
| AS31 | Uzbekistan | *Ae.tauschii* | 15.88 | 27.79 | L | | L |
| AS32 | China | *Ae.tauschii* | 8.92 | 22.90 | L | | H |
| AS33 | Afghanistan | *Ae.tauschii* | 14.32 | 20.83 | L | | H |
| AS34 | N | *Ae.tauschii* | 14.61 | 37.50 | L | | H |
| AS35 | Pakistan | *Ae.tauschii* | 21.06 | 35.17 | L | | L |
| AS36 | N | *Ae.tauschii* | 13.40 | 33.62 | L | | N |
| AS37 | Iran | *Ae.tauschii* | 15.62 | 29.39 | L | | N |
| AS38 | Uzbekistan | *Ae.tauschii* | 12.79 | 26.87 | L | | L |
| AS39 | Pakistan | *Ae.tauschii* | 14.42 | 26.33 | L | | L |
| AS40 | Azerbaijan | *Ae.strangulata* | 15.42 | 23.57 | H | | L |
| AS41 | Afghanistan | *Ae.tauschii* | 14.09 | 27.83 | L | | H |
| AS42 | N | N | 23.41 | 34.35 | L | | N |
| AS43 | N | N | 23.11 | 29.33 | H | | N |
| AS44 | N | N | 14.43 | 24.89 | L | | H |
| AS45 | Iran | *Ae.strangulata* | 14.15 | 37.11 | L | | H |
| AS46 | Pakistan | *Ae.tauschii* | 12.59 | 38.66 | L | | H |
| AS47 | N | N | 13.79 | 18.78 | L | | L |
| AS48 | Azerbaijan | *Ae.strangulata* | 14.83 | 32.71 | H | | H |
| AS49 | Iran | *Ae.tauschii* | 15.91 | 32.33 | L | | H |
| AS50 | 0 | *Ae.strangulata* | 16.44 | 24.72 | L | | L |
| AS51 | Western Asia | *Ae.tauschii* | 16.62 | 21.26 | L | | H |
| AS52 | Pakistan | *Ae.tauschii* | 18.32 | 32.07 | L | | N |
| AS53 | N | N | 14.90 | 32.46 | H | | N |
| AS54 | N | N | 17.74 | 26.83 | L | | L |
| AS55 | India | *Ae.tauschii* | 13.21 | 36.40 | L | | H |
| AS56 | Iran | *Ae.strangulata* | 9.24 | 31.31 | L | | L |
| AS57 | Pakistan | *Ae.tauschii* | 16.84 | 23.30 | L | | H |
| AS58 | Tajikistan | *Ae.tauschii* | 14.08 | 33.27 | L | | L |
| AS59 | N | N | 15.44 | 34.51 | H | | N |
| AS60 | Turkey | *Ae.tauschii* | 14.96 | 33.97 | L | | L |
| AS61 | N | N | 30.14 | 30.52 | H | | H |
| AS62 | Uzbekistan | *Ae.tauschii* | 14.06 | 32.50 | L | | L |
| AS63 | Iran | *Ae.strangulata* | 12.83 | 18.02 | H | | H |
| AS64 | USSR | *Ae.tauschii* | 14.17 | 29.34 | L | | H |
| AS65 | Western Asia | *Ae.tauschii* | 14.58 | 31.46 | L | | L |
| AS66 | Afghanistan | *Ae.tauschii* | 15.11 | 31.98 | H | | L |
| AS67 | N | *Ae.strangulata* | 16.39 | 29.59 | H | | H |
| AS68 | Amercian | *Ae.tauschii* | 26.24 | 33.56 | L | | L |
| AS69 | Azerbaijan | *Ae.strangulata* | 16.84 | 24.62 | H | | L |
| AS70 | Tajikistan | *Ae.tauschii* | 10.75 | 29.09 | L | | H |
| AS71 | Turkey | *Ae.tauschii* | 19.85 | 21.39 | L | | L |
| AS72 | N | N | 14.66 | 28.85 | H | | L |
| AS73 | N | *Ae.strangulata* | 23.32 | 29.58 | L | | H |
| AS74 | Turkey | *Ae.tauschii* | 9.3 | 29.50 | L | | H |
| AS75 | Iran | *Ae.strangulata* | 12.51 | 36.08 | L | | H |
| AS76 | N | N | 25.53 | 23.86 | H | | L |
| AS77 | Turkey | *Ae.tauschii* | 14.06 | 33.19 | L | | H |
| AS78 | N | N | 8.33 | 29.95 | H | | N |
| AS79 | N | N | 8.8 | 30.61 | H | | H |
| AS80 | N | N | 8.23 | 35.12 | L | | H |
| AS81 | N | N | 27.16 | 32.45 | H | | H |
| AS82 | Iran | *Ae.strangulata* | 20.69 | 29.53 | H | | L |
| AS83 | N | N | 23.57 | 30.51 | H | | H |
| AS84 | Tajikistan | *Ae.tauschii* | 16.41 | 33.03 | L | | L |
| AS85 | Uzbekistan | *Ae.tauschii* | 12.93 | 37.90 | L | | L |
| AS86 | Iran | *Ae.strangulata* | 14.84 | 27.10 | L | | H |
| AS87 | Turkey | *Ae.tauschii* | 14.73 | 32.18 | L | | N |
| AS88 | Azerbaijan | *Ae.strangulata* | 13.78 | 28.05 | H | | L |
| AS89 | N | N | 15.03 | 40.53 | H | | L |
| AS90 | Turkey | *Ae.tauschii* | 12.51 | 33.03 | L | | L |
| AS91 | Azerbaijan | *Ae.strangulata* | 15.49 | 25.15 | H | | H |
| AS92 | Iran | *Ae.strangulata* | 15.49 | 30.87 | H | | L |
| AS93 | Iran | *Ae.strangulata* | 19.77 | 34.89 | H | | H |
| AS94 | Pakistan | *Ae.tauschii* | 11.51 | 29.51 | L | | H |
| AS95 | Pakistan | *Ae.tauschii* | 13.08 | 30.74 | L | | H |
| AS96 | Turkey | *Ae.tauschii* | 15.52 | 25.66 | N | | N |
| AS97 | Pakistan | *Ae.tauschii* | 17.82 | 32.93 | L | | H |
| AS98 | Iran | *Ae.strangulata* | 16.32 | 28.74 | H | | H |
| AS99 | N | *Ae.strangulata* | 17.38 | 21.83 | H | | L |
| AS100 | Iran | *Ae.strangulata* | 13.98 | 38.04 | L | | H |
| AS101 | Azerbaijan | *Ae.strangulata* | 13.67 | 35.96 | H | | L |
| AS102 | Iran | *Ae.strangulata* | 14.55 | 28.75 | H | | L |
| AS103 | Azerbaijan | *Ae.strangulata* | 14.97 | 25.18 | H | | L |
| AS104 | Uzbekistan | *Ae.tauschii* | 14.22 | 32.01 | L | | L |
| AS105 | N | *Ae.strangulata* | 14.24 | 27.56 | L | | H |
| AS106 | Iran | *Ae.strangulata* | 13.16 | 27.75 | H | | L |
| AS107 | Turkey | *Ae.strangulata* | 17.13 | 29.13 | L | | H |
| AS108 | Iran | *Ae.strangulata* | 12.79 | 28.20 | H | | H |
| AS109 | N | N | 12.08 | 33.54 | H | | H |
| AS110 | Tajikistan | *Ae.tauschii* | 15.25 | 38.78 | L | | H |
| AS111 | Turkmenistan | *Ae.strangulata* | 11.97 | 23.91 | L | | H |
| AS112 | Azerbaijan | *Ae.strangulata* | 16.01 | 28.11 | N | | H |
| AS113 | Azerbaijan | *Ae.strangulata* | 16.41 | 50.26 | L | | H |
| AS114 | Iran | *Ae.strangulata* | 22.68 | 24.43 | H | | H |
| AS115 | Turkey | *Ae.strangulata* | 11.97 | 26.89 | H | | L |
| AS116 | Armenia/Georgia | *Ae.tauschii* | 12.83 | 28.67 | L | | L |
| AS117 | Iran | *Ae.strangulata* | 11.69 | 38.30 | H | | L |
| AS118 | Afghanistan | *Ae.tauschii* | 15.06 | 35.02 | L | | H |
| AS119 | Pakistan | *Ae.tauschii* | 13.25 | 37.41 | L | | H |
| AS120 | Turkey | *Ae.tauschii* | 14.16 | 22.86 | L | | L |
| AS121 | Iran | *Ae.tauschii* | 14.32 | 30.28 | L | | H |
| AS122 | Iran | *Ae.tauschii* | 12.06 | 27.23 | L | | H |
| AS123 | Iran | *Ae.strangulata* | 10.24 | 19.57 | L | | H |
| AS124 | Iran | *Ae.strangulata* | 28.30 | 28.60 | H | | N |
| AS125 | Iran | *Ae.strangulata* | 12.55 | 41.74 | L | | H |
| AS126 | Iran | *Ae.strangulata* | 12.05 | 22.87 | L | | H |

N represents unknown country/region, subspecies, or genotype. H and L represent the AS2407 homozygous allele and the AS65 homozygous allele, respectively.
